# ﻿Three new species of *Pestalotiopsis* (Amphisphaeriales, Sporocadaceae) were identified by morphology and multigene phylogeny from Hainan and Yunnan, China

**DOI:** 10.3897/mycokeys.107.122026

**Published:** 2024-07-11

**Authors:** Changzhun Yin, Zhaoxue Zhang, Shi Wang, Liguo Ma, Xiuguo Zhang

**Affiliations:** 1 College of Life Sciences, Shandong Normal University, Jinan, 250358, China Shandong Normal University Jinan China; 2 Shandong Provincial Key Laboratory for Biology of Vegetable Diseases and Insect Pests, College of Plant Protection, Shandong Agricultural University, Taian, 271018, China Shandong Agricultural University Taian China; 3 Institute of Plant Protection, Shandong Academy of Agricultural Sciences, Jinan, 250100, China Shandong Academy of Agricultural Sciences Jinan China

**Keywords:** New species, *Pestalotiopsis*, taxonomy

## Abstract

*Pestalotiopsis* fungi are widely distributed all over the world, mainly as plant pathogens, endophytes or saprobes from multiple hosts. In this study, the sequence data analysis based on internal transcribed spacer (ITS), partial beta-tubulin (*tub2*) and partial regions of translation elongation factor 1 alpha (*tef1α*) combined with morphological characteristics was used to identify strains isolated from the diseased leaves of *Aporosadioica* and *Rhaphiolepisindica*, as well as some rotted leaves from Yunnan and Hainan Provinces in China as three new species, viz., *Pestalotiopsisaporosae-dioicae***sp. nov.**, *P.nannuoensis***sp. nov.** and *P.rhaphiolepidis***sp. nov.**

## ﻿Introduction

*Pestalotiopsis* was separated from *Pestalotia* by Steyaert in 1942 and belongs to the Sporocadaceae, Amphisphaeriales, Ascomycota (Steyaert 1949). At present, a total of 420 records of *Pestalotiopsis* have been recorded in the Index Fungorum (http://www.indexfungorum.org/, accessed on 26 Jun 2024). Pestalotioid fungi are a cosmopolitan group of fungi, which have important relationships with different plants as plant pathogens, saprobes or endophytes, and are widely distributed in temperate and tropical regions (Maharachchikumbura et al. 2011, 2012, 2014). As important plant pathogens, pestalotioid species can cause many plant diseases and great economic losses to people (Zhang et al. 2012a; Maharachchikumbura et al. 2013; Jayawardena et al. 2016; Liu et al. 2017; Yang et al. 2017; Diogo et al. 2021; Prasannath et al. 2021). In the past, gray blight disease of tea trees caused by pestalotioid species had caused huge yield losses in southern India (Joshi et al. 2009). In addition, pestalotioid species can also cause the leaf spot of *Taxuschinensis* in China, leaf blight of *Elettariacardamomum* in India, and dieback and stem girdling in young eucalyptus plants in Portugal (Biju et al. 2018; Li et al. 2021; Wang et al. 2021). Therefore, the study of pathogenic pestalotioid species can provide research basis for the treatment and inhibition of diseases and avoid significant economic losses.

At first, *Pestalotiopsis* resembling those taxa having a relationship with *Pestalotia* were also referred to as pestalotioid fungi. Pestalotioid fungi are characterized by multiseptate and fusiform conidia with appendages at one end or both, frequently with some melanized cells. (Bonthond et al. 2018; Liu et al. 2019). Traditionally, pestalotioid species have been classified mainly according to color intensity of the median conidial cell and the hosts (Maharachchikumbura et al. 2014). But the development of DNA based phylogenetic analysis has brought the traditional classification system into dispute. Maharachchikumbura et al. (2014) applied molecular data to the classification of *Pestalotiopsis*. By the difference of multilocus phylogenetic analyses, conidial pigment color, and conidiophores, this group was divided into three genera, *Pestalotiopsis*, *Pseudopestalotiopsis*, and *Neopestalotiopsis*. *Neopestalotiopsis* differs from *Pestalotiopsis* and *Pseudopestalotiopsis* in that two upper median cells are darker than the lowest median cell of the conidia, and its indistinct conidiophores. *Pseudopestalotiopsis* can be easily distinguished from *Pestalotiopsis* due to its three darker median cells. In recent years, many novel species have been introduced into this group by the use of phylogeny approaches together with morphology (Akinsanmi et al. 2017; Liu et al. 2017; Nozawa et al. 2017; Ariyawansa and Hyde 2018; Jiang et al. 2018; Tibpromma et al. 2018; Tsai et al. 2018).

We conducted extensive sampling in southern China to investigate fungal diversity and explore fungal resources. This study aimed to identify *Pestalotiopsis* which was isolated from diseased leaves of *Aporosadioica* and *Rhaphiolepisindica*, as well as some rotted leaves collected from Hainan and Yunnan Provinces by morphological characters and molecular phylogeny, and three new species of *Pestalotiopsis* were described and illustrated.

## ﻿Materials and methods

### ﻿Sample collection and isolation

The isolates used in this study were obtained from diseased or rotted leaves collected in Yunnan and Hainan Provinces from March to May 2023. Cut 5 × 5 mm small square leaves from the fungal infection part of each sample of diseased or rotted leaves and put them into sterile containers respectively. First, immerse all the small square leaves of each sample in 75% ethanol for disinfection for 1 min, and rinse with sterilized water one time after pouring out the ethanol. Then immerse all the small square leaves of each sample in 5% sodium hypochlorite solution for disinfection for 30s, and pour out the sodium hypochlorite solution, rinse them repeatedly with sterilized water three times. After pouring out the sterilized water, pick them up with sterilized tweezers and put them on sterilized filter paper to dry. The sterilized leaves were plated on PDA plates (PDA: 20 g agar, 20 g dextrose, 200 g potato, 1000 ml distilled water, pH 7.0) with sterilized tweezers, then 4 small leaves were placed symmetrically on the surface of each medium, with the disease spot facing down, close to the medium, and the serial number and date were marked on the medium after sealing with a sealing film. The PDA plate was cultured in a constant temperature incubator at 25 °C and the growth of fungi was observed and recorded every day. After 2 to 3 days of culture, the agar with mycelium on the edges of the colony was purified onto a new PDA plate and cultured for 1 to 2 weeks.

### ﻿Morphological and cultural characterization

The PDA plates were photographed on days 7 and 14 with a digital camera (Canon Powershot G7X). The morphological characteristics of fungi were observed with Olympus SZX10 stereomicroscope and Olympus BX53 microscope, then the fungal structures such as conidiomata, conidiophores, conidiogenous cells, conidia, and appendages, were photographed with an Olympus DP80 high-definition color digital camera. The microstructures are measured with the Digimizer software (https://www.digimizer.com/), and the number of samples measured is generally 20–30. All strains were stored in sterilized 10% glycerol at 4 °C. Voucher specimens have been preserved in the Herbarium of the Department of Plant Pathology, Shandong Agricultural University, Taian, China (HSAUP) and Herbarium Mycologicum Academiae Sinicae, Institute of Microbiology, Chinese Academy of Sciences, Beijing, China (HMAS). Ex-holotype living cultures have been preserved in the Shandong Agricultural University Culture Collection (SAUCC). A taxonomy and description of the new species has been uploaded to MycoBank (http://www.mycobank.org/).

### ﻿DNA extraction, PCR amplification, and sequencing

The genomic DNA was extracted from the colonies cultured on PDA by CTAB (cetyl trimethyl ammonium bromide) method and BeaverBeads Plant DNA Kit (Cat. No.: 70409-20; BEAVER Biomedical Engineering Co., Ltd.) (Guo et al. 2000; Wang et al. 2023). Fungal DNA was amplified using polymerase chain reaction (PCR) using three pairs of primers including internal transcribed spacer (ITS), partial beta-tubulin (*tub2*) and partial regions of translation elongation factor 1 alpha (*tef1α*) (White et al. 1990; Glass and Donaldson 1995; O’Donnell et al. 1998; Carbone and Kohn 1999). The reaction was amplified at 25 μL reaction volume, including 12.5 μL 2× Hieff Canace^®^ Plus PCR Master Mix (Shanghai, China) (with dye) (Yeasen Biotechnology, Shanghai, China, Cat No. 10154ES03), 1 μL forward primer, 1 μL reverse primer, and 1 μL genomic DNA template, add distilled deionized water to a total volume 25 μL. The PCR amplification products were detected by electrophoresis in 2% agarosegels and the amplification effect was determined by observing the fragments with UV light (Zhang et al. 2022). Then use a Gel Extraction Kit (Cat: AE0101-C) (Shandong Sparkjade Biotechnology Co., Ltd.) for gel recovery. DNA sequencing and primer synthesis were completed by Tsingke Biotechnology Co., Ltd. (Qingdao, China). The bidirectional sequencing results of the three primers were examined and spliced using MEGA v. 7.0 (Kumar et al. 2016). The sequences of all three new species have been uploaded to GenBank, and the Genebank numbers of all strain sequences used in this study are shown in Table 1.

**Table 1.** GenBank numbers used in the phylogenetic analysis of *Pestalotiopsis*.

Species	Isolate	Origin	Substrate	GenBank accession			References
				ITS	*tub2*	*tef1α*	
*Neopestalotiopsismagna*	MFLUCC 12-0652*	France	*Pteridium* sp.	KF582795	KF582793	KF582791	(Maharachchikumbura et al. 2014)
*Pestalotiopsisabietis*	CFCC 53013	China	*Abiesfargesii*	MK397015	MK622282	MK622279	(Gu et al. 2021)
	CFCC 53011*	China	*Abiesfargesii*	MK397013	MK622280	MK622277	
	CFCC 53012	China	*Abiesfargesii*	MK397014	MK622281	MK622278	
*P.adusta*	MFLUCC 10-146	Thailand	*Syzygium* sp.	JX399007	JX399038	JX399071	(Maharachchikumbura et al. 2012)
	ICMP 6088*	Fiji	Refrigerator door	JX399006	JX399037	JX399070	
*P.aggestorum*	LC8186	China	*Camelliasinensis*	KY464140	KY464160	KY464150	(Liu et al. 2017)
	LC6301*	China	*Camelliasinensis*	KX895015	KX895348	KX895234	
*P.anhuiensis*	CFCC 54791*	China	*Cyclobalanopsisglauca*	ON007028	ON005056	ON005045	(Jiang et al. 2022b)
*P.anacardiacearum*	IFRDCC 2397*	China	*Mangiferaindica*	KC247154	KC247155	KC247156	(Sajeewa et al. 2013)
*P.arengae*	CBS 331.92*	Singapore	*Arengaundulatifolia*	KM199340	KM199426	KM199515	(Maharachchikumbura et al. 2014)
*P.arceuthobii*	CBS 434.65*	USA	*Arceuthobiumcampylopodum*	KM199341	KM199427	KM199516	(Maharachchikumbura et al. 2014)
***P.aporosae-dioicae***	SAUCC224004*	**China**	***Aporosadioica***	**OR733506**	**OR912985**	**OR912988**	This study
	**SAUCC224005**	**China**	***Aporosadioica***	**OR733505**	**OR912986**	**OR912989**	
*P.appendiculata*	CGMCC 3.23550*	China	*Rhododendrondecorum*	OP082431	OP185516	OP185509	(Gu et al. 2022)
*P.australis*	CBS 114193*	New South Wales	*Grevillea* sp.	KM199332	KM199383	KM199475	(Maharachchikumbura et al. 2014)
	CBS 111503	South Africa	*Proteaneriifolia*	KM199331	KM199382	KM199557	
*P.australasiae*	CBS 114141	New South Wales	*Protea* sp.	KM199298	KM199410	KM199501	(Maharachchikumbura et al. 2014)
	CBS 114126*	New Zealand	*Knightia* sp.	KM199297	KM199409	KM199499	
*P.biciliata*	CBS 236.38	Italy	*Paeonia* sp.	KM199309	KM199401	KM199506	(Maharachchikumbura et al. 2014)
	CBS 124463*	Slovakia	*Platanushispanica*	KM199308	KM199399	KM199505	
*P.brachiata*	LC2988*	China	*Camellia* sp.	KX894933	KX895265	KX895150	(Liu et al. 2017)
	LC8188	China	*Camellia* sp.	KY464142	KY464162	KY464152	
*P.brassicae*	CBS 170.26*	New Zealand	*Brassicanapus*	KM199379	NA	KM199558	(Maharachchikumbura et al. 2014)
*P.camelliae*	MFLUCC 12-0277*	China	*Camelliajaponica*	JX399010	JX399041	JX399074	(Zhang et al. 2012b)
*P.camelliae-oleiferae*	CSUFTCC08*	China	*Camelliaoleifera*	OK493593	OK562368	OK507963	(Li et al. 2021)
	CSUFTCC09	China	*Camelliaoleifera*	OK493594	OK562369	OK507964	
*P.cangshanensis*	CGMCC 3.23544*	China	*Rhododendrondelavayi*	OP082426	OP185517	OP185510	(Gu et al. 2022)
*P.castanopsidis*	CFCC 54430*	China	*Castanopsislamontii*	OK339732	OK358508	OK358493	(Jiang et al. 2022b)
*P.chamaeropis*	CBS 186.71*	Italy	*Chamaeropshumilis*	KM199326	KM199391	KM199473	(Maharachchikumbura et al. 2012)
*P.changjiangensis*	CFCC 54314*	China	*Castanopsistonkinensis*	OK339739	OK358515	OK358500	(Jiang et al. 2022b)
	CFCC 52803	China	*Cyclobalanopsis* sp.	OK339741	OK358517	OK358502	
	CFCC 54433	China	*Castanopsishainanensis*	OK339740	OK358516	OK358501	
*P.chiangmaiensis*	MFLU 22-0164*	Thailand	*Phyllostachysedulis*	OP497990	OP752137	OP753374	(Sun et al. 2023)
*P.chiaroscuro*	BRIP 72970*	Australia	*Sporobolusnatalensis*	OK422510	OK423752	OK423753	(Crous et al. 2022)
*P.chinensis*	MFLUCC 12-0273	China	*Taxus* sp.	JX398995	NA	NA	(Maharachchikumbura et al. 2012)
*P.clavata*	MFLUCC 12-0268*	China	*Buxus* sp.	JX398990	JX399025	JX399056	(Maharachchikumbura et al. 2012)
*P.colombiensis*	CBS 118553*	Colombia	*Eucalyptusurograndis*	KM199307	KM199421	KM199488	(Maharachchikumbura et al. 2014)
*P.cyclobalanopsidis*	CFCC 54328*	China	*Cyclobalanopsisglauca*	OK339735	OK358511	OK358496	(Jiang et al. 2022b)
	CFCC 55891	China	*Cyclobalanopsisglauca*	OK339736	OK358512	OK358497	
*P.daliensis*	CGMCC 3.23548*	China	*Rhododendrondecorum*	OP082429	OP185518	OP185511	(Gu et al. 2022)
*P.dianellae*	CPC 32261	Australia	*Dianella* sp.	MG386051	MG386164	NA	(Crous et al. 2017)
*P.digitalis*	MFLU 14-0208*	New Zealand	*Digitalispurpurea*	KP781879	KP781883	NA	(Liu et al. 2015)
*P.dilucida*	LC3232*	China	*Camelliasinensis*	KX894961	KX895293	KX895178	(Liu et al. 2017)
	LC8184	China	*Camelliasinensis*	KY464138	KY464158	KY464148	
*P.diploclisiae*	CBS 115449	China	*Psychotriatutcheri*	KM199314	KM199416	KM199485	(Maharachchikumbura et al. 2014)
	CBS 115587*	China	*Diploclisiaglaucescens*	KM199320	KM199419	KM199486	
*P.disseminata*	CBS 143904	New Zealand	*Perseaamericana*	MH554152	MH554825	MH554587	(Liu et al. 2017)
*P.diversiseta*	MFLUCC 12-0287*	China	*Rhododendron* sp.	JX399009	JX399040	JX399073	(Maharachchikumbura et al. 2012)
*P.doitungensis*	MFLUCC 14-0090*	Thailand	*Dendrobium* sp.	MK993574	MK975837	MK975832	(Ma et al. 2019)
*P.dracontomelonis*	MFLU 14-0207*	Thailand	*Dracontomelon* sp.	KP781877	NA	KP781880	(Liu et al. 2015)
*P.dracaenae*	HGUP 4037*	China	*Dracaenafragrans*	MT596515	MT598645	MT598644	(Ariyawansa et al. 2015)
*P.dracaenicola*	MFLUCC 18-0913*	Thailand	*Dracaena* sp.	MN962731	MN962733	MN962732	(Chaiwan et al. 2020)
*P.eleutherococci*	HMJAU 60189*	China	*Eleutherococcusbrachypus*	NR182556	NA	NA	(Tian et al. 2022)
*P.endophytica*	MFLU 20-0607*	Thailand	*Magnoliagarrettii*	MW263946	NA	MW417119	(De Silva et al. 2021)
*P.ericacearum*	IFRDCC 2439*	China	*Rhododendrondelavayi*	KC537807	KC537821	KC537814	(Zhang et al. 2013)
*P.etonensi*s	BRIP 66615*	Australia	*Sporobolusjacquemontii*	MK966339	MK977634	MK977635	(Crous et al. 2020)
*P.ficicola*	SAUCC230046*	China	*Ficusmicrocarpa*	OQ691974	OQ718749	OQ718691	(Zhang et al. 2023)
*P.foliicola*	CFCC 57359	China	*Castanopsisfaberi*	ON007030	ON005058	ON005047	(Jiang et al. 2022b)
	CFCC 57360	China	*Castanopsisfaberi*	ON007031	ON005059	ON005048	
	CFCC 54440*	China	*Castanopsisfaberi*	ON007029	ON005057	ON005046	
*P.furcat*a	MFLUCC 12-0054*	Thailand	*Camelliasinensis*	JQ683724	JQ683708	JQ683740	(Watanabe et al. 2018)
*P.fusoidea*	CGMCC 3.23545*	China	*Rhododendrondelavayi*	OP082427	OP185519	OP185512	(Gu et al. 2022)
*P.formosana*	NTUCC 17-009*	China	Poaceae sp.	MH809381	MH809385	MH809389	(Akinsanmi et al. 2017)
*P.gaultheriae*	IFRD 411-014*	China	*Gaultheriaforrestii*	KC537805	KC537819	KC537812	(Maharachchikumbura et al. 2014)
*P.gibbosa*	NOF 3175*	Canada	*Gaultheriashallon*	LC311589	LC311590	LC311591	(Watanabe et al. 2018)
*P.grandis-urophylla*	E-72-02	Brazil	*Eucalyptus* sp.	KU926708	KU926716	KU926712	(Carvalho et al. 2019)
	E-72-03	Brazil	*Eucalyptus* sp.	KU926709	KU926717	KU926713	
	E-72-04	Brazil	*Eucalyptus* sp.	KU926710	KU926718	KU926714	
	E-72-06	Brazil	*Eucalyptus* sp.	KU926711	KU926719	KU926715	
*P.guangdongensis*	ZHKUCC 22-0016*	China	*Arengapinnata*	ON180762	ON221548	ON221520	(Xiong et al. 2022)
*P.guangxiensis*	CFCC 54308*	China	*Quercusgriffithii*	OK339737	OK358513	OK358498	(Jiang et al. 2022b)
	CFCC 54300	China	*Quercusgriffithii*	OK339738	OK358514	OK358499	
*P.grevilleae*	CBS 114127*	Australia	*Grevillea* sp.	KM199300	KM199407	KM199504	(Maharachchikumbura et al. 2014)
*P.guizhouensis*	CFCC 54803	China	*Cyclobalanopsisglauca*	ON007035	ON005063	ON005052	(Jiang et al. 2022b)
	CFCC 57364	China	*Cyclobalanopsisglauca*	ON007036	ON005064	ON005053	
*P.hawaiiensis*	CBS 114491*	USA	*Leucospermum* sp.	KM199339	KM199428	KM199514	(Maharachchikumbura et al. 2014)
*P.hispanica*	CBS 115391	Portugal	*Eucalyptusglobulus*	MH553981	MH554640	MH554399	(Maharachchikumbura et al. 2014)
*P.hollandica*	CBS 265.33*	The Nethelands	*Sciadopitysverticillata*	KM199328	KM199388	KM199481	(Maharachchikumbura et al. 2014)
*P.humicola*	CBS 336.97*	Papua New Guinea	Soil	KM199317	KM199420	KM199484	(Maharachchikumbura et al. 2014)
*P.hunanensis*	CSUFTCC18	China	*Camelliaoleifera*	OK493600	OK562375	OK507970	(Li et al. 2021)
	CSUFTCC15*	China	*Camelliaoleifera*	OK493599	OK562374	OK507969	
*P.hydei*	MFLUCC 20-0135	Thailand	*Litseaelliptica*	MW266063	MW251112	MW251113	(Huanaluek et al. 2021)
*P.iberica*	CAA 1005	Spain	*Pinussylvestris*	MW732250	MW759034	MW759037	(Monteiro et al. 2021)
	CAA 1006	Spain	*Pinusradiata*	MW732249	MW759036	MW759039	
	CAA 1004*	Spain	*Pinusradiata*	MW732248	MW759035	MW759038	
*P.intermedia*	MFLUCC 12-0259*	China	Unidentified tree	JX398993	JX399028	JX399059	(Maharachchikumbura et al. 2012)
*P.inflexa*	MFLUCC 12-0270*	China	Unidentified tree	JX399008	JX399039	JX399072	(Maharachchikumbura et al. 2012)
*P.italiana*	MFLU 14-0214*	Italy	*Cupressusglabra*	KP781878	KP781882	KP781881	(Liu et al. 2015)
*P.jesteri*	CBS 109350*	Papua New Guinea	*Fragraeabodenii*	KM199380	NA	KM199554	(Maharachchikumbura et al. 2014)
*P.jiangxiensis*	LC4399*	China	*Camellia* sp.	KX895009	KX895341	KX895227	(Liu et al. 2017)
*P.jiangsuensis*	CFCC 59538	China	*Pinusmassoniana*	OR533577	OR539191	OR539186	(Li et al. 2024)
*P.jinchanghensis*	LC8190	China	*Camelliasinensis*	KY464144	KY464164	KY464154	(Liu et al. 2017)
	LC6636*	China	*Camelliasinensis*	KX895028	KX895361	KX895247	
*P.kandelicola*	NCYUCC 19-0354	China	*Kandeliacandel*	MT560723	MT563100	MT563102	(Hyde et al. 2020)
	NCYUCC 19-0355*	China	*Kandeliacandel*	MT560722	MT563099	MT563101	
*P.kaki*	KNU-PT-1804*	Korea	*Diospyroskaki*	LC552953	LC552954	LC553555	(Das et al. 2020)
*P.kenyana*	LC6633	China	*Camelliasinensis*	KX895027	KX895360	KX895246	(Maharachchikumbura et al. 2014)
	CBS 442.67*	Kenya	*Coffea* sp.	KM199302	KM199395	KM199502	
*P.knightiae*	CBS 114138*	New Zealand	*Knightia* sp.	KM199310	KM199408	KM199497	(Maharachchikumbura et al. 2014)
	CBS 111963	New Zealand	*Knightia* sp.	KM199311	KM199406	KM199495	
*P.krabiensis*	MFLUCC 16-0260*	Thailand	*Pandanus* sp.	MH388360	MH412722	MH388395	(Tibpromma et al. 2018)
*P.leucadendri*	CBS 121417*	South Africa	*Leucadendron* sp.	MH553987	MH554654	MH554412	(Liu et al. 2019)
*P.licualicola*	HGUP 4057*	China	*Licualagrandis*	KC492509	KC481683	KC481684	(Geng et al. 2013)
*P.lijiangensis*	CFCC 50738*	China	*Castanopsiscarlesii*	KU860520	NA	NA	(Zhou et al. 2018)
*P.linearis*	MFLUCC 12-0271*	China	*Trachelospermum* sp.	JX398992	JX399027	JX399058	(Maharachchikumbura et al. 2012)
*P.linguae*	ZHKUCC 22-0159	China	*Pyrrosialingua*	OP094104	OP186108	OP186110	(Li et al. 2023)
*P.lithocarpi*	CFCC 55893	China	*Lithocarpuschiungchungensis*	OK339743	OK358519	OK358504	(Jiang et al. 2022b)
	CFCC 55100*	China	*Lithocarpuschiungchungensis*	OK339742	OK358518	OK358503	
*P.longiappendiculata*	LC3013*	China	*Camelliasinensis*	KX894939	KX895271	KX895156	(Liu et al. 2017)
*P.loeiana*	MFLU 22-0167*	Thailand	Unidentified tree	OP497988	OP713769	OP737881	(Sun et al. 2023)
*P.lushanensis*	LC8182	China	*Camellia* sp.	KY464136	KY464156	KY464146	(Liu et al. 2017)
	LC8183	China	*Camellia* sp.	KY464137	KY464157	KY464147	
	LC4344*	China	*Camellia* sp.	KX895005	KX895337	KX895223	
*P.macadamiae*	BRIP 63739b	Australia	*Macadamiaintegrifolia*	KX186587	KX186679	KX186620	(Akinsanmi et al. 2017)
	BRIP 63741a	Australia	*Macadamiaintegrifolia*	KX186586	KX186678	KX186619	
	BRIP 63738b*	Australia	*Macadamiaintegrifolia*	KX186588	KX186680	KX186621	
*P.malayana*	CBS 102220*	Malaysia	*Macarangatriloba*	KM199306	KM199411	KM199482	(Maharachchikumbura et al. 2014)
*P.manyueyuanensis*	NTUPPMCC 18-165*	Taiwan	*Ophocordyceps* sp.	OR125060	OR126306	OR126313	(Hsu et al. 2024)
*P.menhaiensis*	CGMCC 3.18250*	China	*Ophocordyceps* sp.	KU252272	KU252488	KU252401	(Li et al. 2024)
*P.microspora*	SS1-033I	Canada	*Cornuscanadensis*	MT644300	NA	NA	(Zhao and Li 1995)
*P.montellica*	MFLUCC 12-0279	China	dead plant material	JX399012	JX399043	JX399076	(Maharachchikumbura et al. 2012)
*P.monochaeta*	CBS 144.97*	The Nethelands	*Quercusrobur*	KM199327	KM199386	KM199479	(Maharachchikumbura et al. 2014)
	CBS 440.83	The Nethelands	*Taxusbaccata*	KM199329	KM199387	KM199480	
*P.multicolor*	CFCC59981	China	*Taxuschinensis*	OQ626676	OQ714336	OQ714341	(Wang et al. 2024)
*P.nanjingensis*	CSUFTCC16*	China	*Camelliaoleifera*	OK493602	OK562377	OK507972	(Li et al. 2021)
*P.nanningensis*	CSUFTCC10*	China	*Camelliaoleifera*	OK493596	OK562371	OK507966	(Li et al. 2021)
***P.nannuoensis***	SAUCC232203*	**China**	**Unknown host**	**OR733504**	**OR863909**	**OR912991**	This study
	**SAUCC232204**	**China**	**Unknown host**	**OR733503**	**OR863910**	**OR912992**	
*P.novae-hollandiae*	CBS 130973*	Australia	*Banksiagrandis*	KM199337	KM199425	KM199511	(Maharachchikumbura et al. 2014)
*P.neolitseae*	NTUCC 17-011*	China	*Neolitseavillosa*	MH809383	MH809387	MH809391	(Akinsanmi et al. 2017)
*P.oryzae*	CBS 171.26	Italy	Unknown host	KM199304	KM199397	KM199494	(Maharachchikumbura et al. 2014)
	CBS 353.69*	Denmark	*Oryzasativa*	KM199299	KM199398	KM199496	
	CBS 111522	USA	*Telopea* sp.	KM199294	KM199394	KM199493	
*P.pallidotheae*	MAFF 240993*	Japan	*Pierisjaponica*	AB482220	NA	NA	(Watanabe et al. 2010)
*P.pandanicola*	MFLUCC 16-0255*	Thailand	*Pandanus* sp.	MH388361	MH412723	MH388396	(Tibpromma et al. 2018)
*P.papuana*	CBS 331.96*	Papua New Guinea	Coastal soil	KM199321	KM199413	KM199491	(Maharachchikumbura et al. 2014)
	CBS 887.96	Papua New Guinea	*Cocosnucifera*	KM199318	KM199415	KM199492	
*P.parva*	CBS 278.35	Thailand	*Delonixregia*	KM199313	KM199405	KM199509	(Maharachchikumbura et al. 2014)
	CBS 265.37*	Thailand	*Delonixregia*	KM199312	KM199404	KM199508	
*P.phoebes*	SAUCC230093*	China	*Phoebezhennan*	OQ692028	OQ718803	OQ718745	(Zhang et al. 2023)
*P.pini*	MEAN 1092	Portugal	*Pinuspinea*	MT374680	MT374705	MT374693	(Silva et al. 2020)
*P.photiniicola*	GZCC 16-0028*	China	*Photiniaserrulata*	KY092404	KY047663	KY047662	(Chen et al. 2017)
*P.pinicola*	KUMCC 19-0183*	China	*Pinusarmandii*	MN412636	MN417507	MN417509	(Tibpromma et al. 2019)
*P.portugallica*	CBS 393.48*	Portugal	Unknown host	KM199335	KM199422	KM199510	(Maharachchikumbura et al. 2014)
***P.rhaphiolepis***	SAUCC367701*	**China**	***Rhaphiolepisindica***	**OR733502**	**OR863906**	**OR912994**	This study
	**SAUCC367702**	**China**	***Rhaphiolepisindica***	**OR733501**	**OR863907**	**OR912995**	
*P.rhizophorae*	MFLUCC 17-0416*	Thailand	*Rhizophoramucronata*	MK764283	MK764349	MK764327	(Norphanphoun et al. 2019)
*P.rhodomyrti*	HGUP4230*	China	*Rhodomyrtustomentosa*	KF412648	KF412642	KF412645	(Song et al. 2013)
*P.rhododendri*	IFRDCC 2399*	China	*Rhododendronsinogrande*	KC537804	KC537818	KC537811	(Zhang et al. 2013)
*P.rosea*	MFLUCC 12-0258*	China	*Pinus* sp.	JX399005	JX399036	JX399069	(Maharachchikumbura et al. 2012)
*P.rosarioides*	CGMCC 3.23549*	China	*Rhododendrondecorum*	OP082430	OP185520	OP185513	(Gu et al. 2022)
*P.sabal*	ZHKUCC 22-0035*	China	*Sabalmexicana*	ON180775	ON221561	ON221533	(Xiong et al. 2022)
*P.sequoiae*	MFLUCC 13-0399*	Italy	*Sequoiasempervirens*	KX572339	NA	NA	(Li et al. 2016)
*P.scoparia*	CBS 176.25*	China	*Chamaecyparis* sp.	KM199330	KM199393	KM199478	(Maharachchikumbura et al. 2014)
*P.shaanxiensis*	CFCC 57356	China	*Quercusvariabilis*	ON007027	ON005055	ON005044	(Jiang et al. 2022b)
	CFCC 54958*	China	*Quercusvariabilis*	ON007026	ON005054	ON005043	
*P.shoreae*	MFLUCC 12-0314*	Thailand	*Shoreaobtusa*	KJ503811	KJ503814	KJ503817	(Song et al. 2014)
*P.sichuanensis*	CGMCC 3.18244*	China	*Camelliasinensis*	KX146689	KX146807	KX146748	(Wang et al. 2019)
*P.silvicola*	CFCC 57363	China	*Cyclobalanopsiskerrii*	ON007034	ON005062	ON005051	(Jiang et al. 2022b)
*P.silvicola*	CFCC 55296*	China	*Cyclobalanopsiskerrii*	ON007032	ON005060	ON005049	(Jiang et al. 2022b)
	CFCC 54915	China	*Cyclobalanopsiskerrii*	ON007033	ON005061	ON005050	
*P.smilacicola*	MFLU 22-0165*	Thailand	*Smilax* sp.	OP497991	OP762673	OP753376	(Sun et al. 2023)
*P.sonneratiae*	CFCC 57394*	China	*Sonneratiaapetala*	ON114184	ON086816	ON086812	(Jiang et al. 2022a)
*P.spatholobi*	SAUCC231201*	China	*Spatholobussuberectus*	OQ692023	OQ718798	OQ718740	(Zhang et al. 2023)
*P.spathuliappendiculata*	CBS 144035*	Australia	*Phoenixcanariensis*	MH554172	MH554845	MH554607	(Liu et al. 2019)
*P.spathulata*	CBS 356.86*	Chile	*Gevuinaavellana*	KM199338	KM199423	KM199513	(Maharachchikumbura et al. 2014)
*P.suae*	CGMCC 3.23546*	China	*Rhododendrondelavayi*	OP082428	OP185521	OP185514	(Gu et al. 2022)
*P.taxicola*	CFCC59976	China	*Taxuschinensis*	OQ626673	OQ714333	OQ714338	(Wang et al. 2024)
*P.telopeae*	CBS 113606	Australia	*Telopea* sp.	KM199295	KM199402	KM199498	(Maharachchikumbura et al. 2014)
	CBS 114161*	Australia	*Telopea* sp.	KM199296	KM199403	KM199500	
	CBS 114137	Australia	*Protea* sp.	KM199301	KM199469	KM199559	
*P.thailandica*	MFLUCC 17-1616*	Thailand	*Rhizophoramucronata*	MK764285	MK764351	MK764329	(Norphanphoun et al. 2019)
*P.terricola*	CBS 141.69*	Pacific islands	Soil	MH554004	MH554680	MH554438	(Liu et al. 2019)
*P.trachicarpicola*	OP068*	China	*Trachycarpusfortunei*	JQ845947	JQ845945	JQ845946	(Zhang et al. 2012a)
*P.trachycarpicola*	BJFUCC42	China	*Taxuschinensis*	OQ626674	OQ714334	OQ714339	(Zhang et al. 2012a)
*P.tumida*	CFCC 55158*	China	*Rosachinensis*	OK560610	OM158174	OL814524	(Peng et al. 2022)
*P.unicolor*	MFLUCC 12-0275	China	Unidentified tree	JX398998	JX399029	JX399063	(Maharachchikumbura et al. 2012)
	MFLUCC 12-0276*	China	*Rhododendron* sp.	JX398999	JX399030	NA	
*P.verruculosa*	MFLUCC 12-0274*	China	*Rhododendron* sp.	JX398996	NA	JX399061	(Maharachchikumbura et al. 2012)
*P.yunnanensis*	HMAS 96359*	China	*Podocarpusmacrophyllus*	AY373375	NA	NA	(Wei et al. 2013)
*P.yanglingensis*	LC3412	China	*Camelliasinensis*	KX894980	KX895312	KX895197	(Liu et al. 2017)
	LC4553*	China	*Camelliasinensis*	KX895012	KX895345	KX895231	

Notes: New species established in this study are shown in bold. Those marked “*” in the table are represented as ex-type or ex-epitype strains. NA: Not available.

### ﻿Phylogenetic analyses

According to the latest publication of this genus, the reference sequences used in this study (Table 1) were obtained from the National Center for Biotechnology Information (NCBI) (Li et al. 2024). Reference sequences and sequences obtained from the sequenced strains were aligned and manually corrected by MAFFT 7 online service with the Auto strategy (http://mafft.cbrc.jp/alignment/server/) (Katoh et al. 2019). Based on maximum likelihood (ML) and Bayesian inference (BI) algorithms, the phylogenetic analysis of multilabel data was carried out. Run ML and BI on the CIPRES Science Gateway portal (https://www.phylo.org/) (Miller et al. 2012). ML was performed on RaxML-HPC2 of XSEDE (8.2.12) (Stamatakis 2014), 1000 fast bootstrap repeats were performed using GTRGAMMA model of nucleotide evolution. MrModeltest v.2.3 (Nylander 2001) is used to screen the optimal evolutionary model, and BI was performed on XSEDE (3.2.7a) (Huelsenbeck and Ronquist 2001; Ronquist and Huelsenbeck 2012; Ronquist et al. 2012). When the mean standard deviation of separation frequency is less than 0.01, output the topology. View and adjust phylogenetic trees in FigTree v. 1.4.4 (http://tree.bio.ed.ac.uk/software/figtree) and beautify the phylogenetic trees with Adobe Illustrator CC 2019. The names of the isolates in this study are marked in red in the phylogenetic tree.

## ﻿Result

### ﻿Phylogenetic analyses

**Figure 1.**
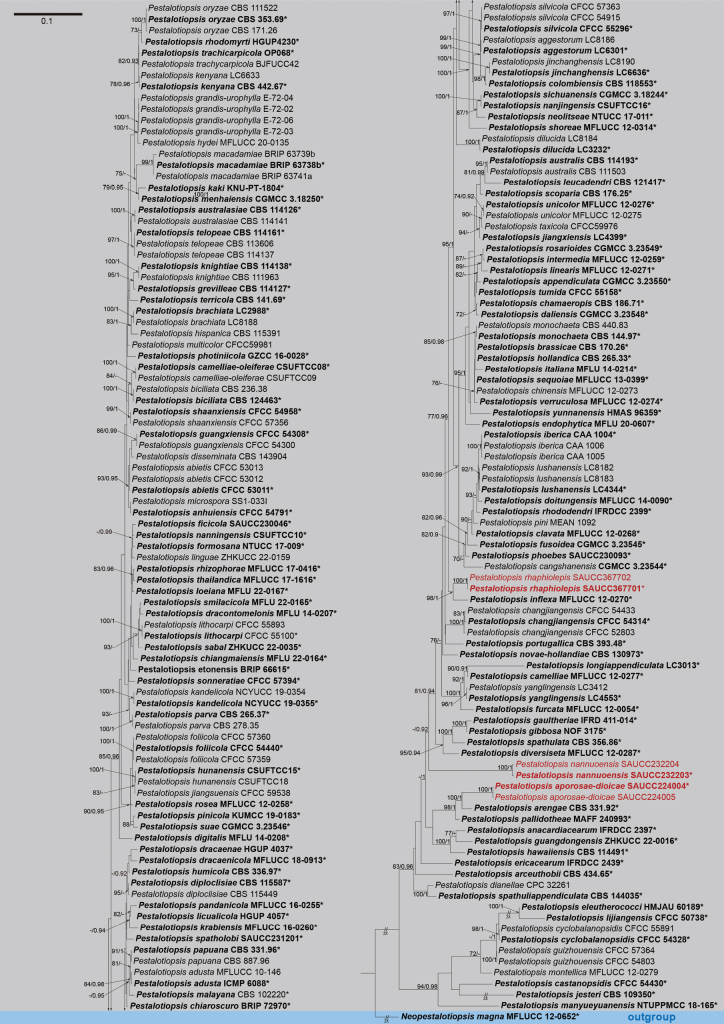
A Maximum Likelihood phylogram of *Pestalotiopsis* based on ITS, *tub2* and *tef1α* gene sequences, and MFLUCC 12-0652 of *Neopestalotiopsismagna* as the tree root of *Pestalotiopsis*. The Maximum Likelihood Bootstrap Value (left, MLBV≥70%) and Bayesian Inference Posterior Probability (right, BIPP≥0.90), separated by a slash line, are marked at the node. The scale bar at the top left represents 0.1 nucleotide changes at each site. Some shortened branches are represented by double slashes and the number of fold times. The strains in this study are shown in red.

By analyzing the sequence data sets of ITS, *tub2* and *tef1α*, the interspecific relationships of *Pestalotiopsis* were inferred. The phylogenetic analysis of *Pestalotiopsis* strains contained 183 sequences, using *Neopestalotiopsismagna* (MFLUCC 12-0652) as the outgroup. A total of 1579 characters including gaps (523 of ITS, 530 of *tub2* and 526 of *tef1α*) were included in the phylogenetic analysis. There were 915 constant, 185 variable but parsimony non-informative, and 479 parsimony informative characters. In Bayesian inference, GTR + I + G is used as the optimal evolutionary model of ITS and *tub2*, and HKY + I + G is used as the optimal evolutionary model of *tef1α*. The final ML optimization likelihood was -15175.820563. The trees obtained by the ML and BI methods are similar, and the ML tree with the best score was shown in Fig. 1, the Maximum Likelihood Bootstrap Values and Bayesian Inference Posterior Probabilities (MLBS/BIPP) are marked at the node position of the phylogenetic tree. On the basis of previous studies, six strains of *Pestalotiopsis* were imported into the phylogenetic analysis in this study. The six new strains introduced in this study were divided into three monophyletic branches in the phylogenetic tree, representing three new species of *Pestalotiopsis*, *P.aporosae-dioicae* sp. nov., *P.nannuoensis* sp. nov. and *P.rhaphiolepidis* sp. nov. Finally, the 183 strains were divided into 135 species clades in the phylogenetic map.

### ﻿Taxonomy

#### ﻿ Pestalotiopsis aporosae-dioicae

Taxon classificationFungiAmphisphaerialesSporocadaceae

C.Z. Yin, Z.X. Zhang & X.G. Zhang sp. nov.

91FDF8F2-AB08-5B3A-8B55-601A5E70EE22

 851279

Fig. 2

##### Type.

China, Yunnan Province, Jinghong City, Sancha River (22°10'10"N, 100°51'49"E), from diseased leaves of *Aporosadioica*, 19 Mar 2023, C.Z. Yin, Z.X. Zhang and X.G. Zhang, holotype HMAS 352667, ex-type living culture SAUCC224004.

**Figure 2.**
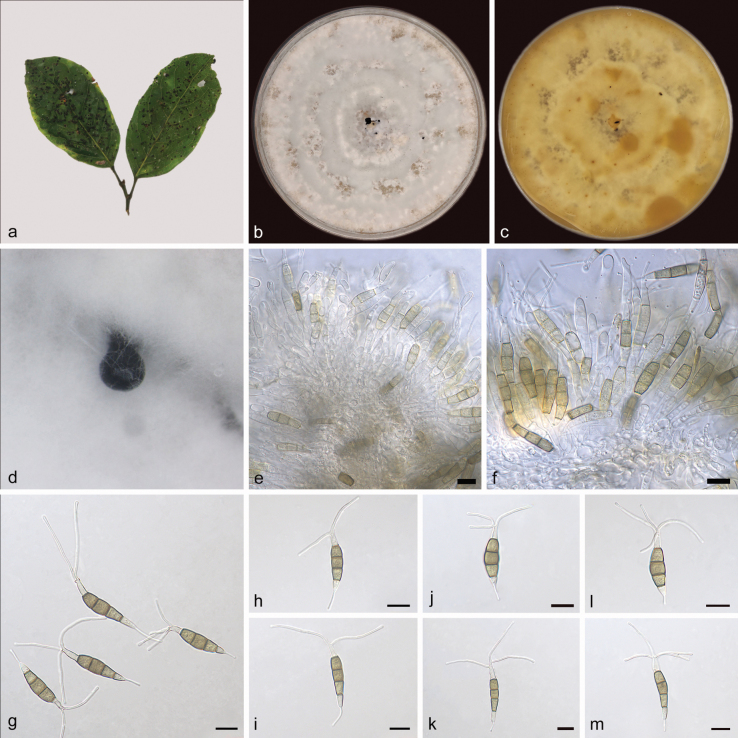
*Pestalotiopsisaporosae-dioicae* (holotype: HMAS 352667) **a** leaves of host *Aporosadioica***b, c** the front and back of the colony after 14 days of culture on PDA **d** conidiomata on PDA **e, f** conidiophores and conidiogenous cells **g–m** conidia. Scale bars: 10 μm (**e–m**).

##### Etymology.

Referring to the name of the host plant *Aporosadioica*.

##### Description.

Conidiomata in culture on PDA, 600–1000 µm diam, globular, solitary, black conidial masses permeated above the mycelium. Conidiophores mostly degenerated into conidiogenous cells, hyaline. Conidiogenous cells smooth, clavate, hyaline, aggregative, 16.1–22.2 × 3.9–5.5 μm. Conidia fusiform, 4-septate, slightly curved or straight, 25.6–35.2 × 5.0–7.1 μm; basal cell conical, hyaline, rough, thin-walled, 3.9–9.7 µm; three median cells subcylindrical, light brown or brown, rough, thick-walled, the first median cell from base 4.9–7.0 μm, the second median cell 4.8–7.0 μm, the third median cell 4.6–6.9 μm, together 14.9–20.2 μm; apical cell subcylindrical, hyaline, smooth, thin–walled, 4.7–8.3 µm; basal appendage tubular, single, centric, straight or slightly bent, unbranched, 4.0–13.2 µm; apical appendages tubular, 2–4, straight or bent, unbranched, 8.8–31.7 μm. Sexual morph not observed.

##### Culture characteristics.

After 14 days of dark cultivation at 25 °C on PDA, the colony diameter reached 90 mm, and the growth rate is 6.2–6.6 mm/day. Colonies filamentous to circular, aerial mycelium on surface raised, white, dense, forms multiple rings from the middle to the edge, fruiting bodies black; reverse yellow, brown in parts.

##### Additional specimen examined.

China, Yunnan Province, Jinghong City, Sancha River, from diseased leaves of *Aporosadioica*, 19 Mar. 2023, C.Z. Yin, Z.X. Zhang and X.G. Zhang, living culture SAUCC224005.

##### Notes.

According to phylogenetic trees based on ITS, *tub2* and *tef1α*, *Pestalotiopsisaporosae-dioicae* sp. nov. was closely related to *P.arengae* in a well support branch (ML/BI = 100/1). *P.aporosae-dioicae* was different from *P.arengae* by 14/508 bp in ITS, 51/529 bp in *tub2*, and 10/465 bp in *tef1α*. Morphologically, *P.aporosae-dioicae* was different from *P.arengae* by having thinner conidia (*P.aporosae-dioicae*: 25.6–35.2 × 5.0–7.1 vs. *P.arengae*: 25.0–32.0 × 7.0–9.5 µm) and longer basal appendages (*P.aporosae-dioicae*: 4.0–13.2 vs. *P.arengae*: 1.5–3.0 μm) (Maharachchikumbura et al. 2014). Therefore, *Pestalotiopsisaporosae-dioicae* was identified as a new species of *Pestalotiopsis* by morphological and phylogenetic comparison.

#### ﻿ Pestalotiopsis nannuoensis

Taxon classificationFungiAmphisphaerialesSporocadaceae

C.Z. Yin, Z.X. Zhang & X.G. Zhang sp. nov.

A56A7BFE-5526-501D-947F-01582D530223

 851280

Fig. 3

##### Type.

China, Yunnan Province, Menghai County, Nannuo Mountain (21°55'25"N, 100°35'41"E), from rotted leaves, 18 Mar 2023, C.Z. Yin, Z.X. Zhang and X.G. Zhang, holotype HMAS 352668, ex-type living culture SAUCC232203.

**Figure 3.**
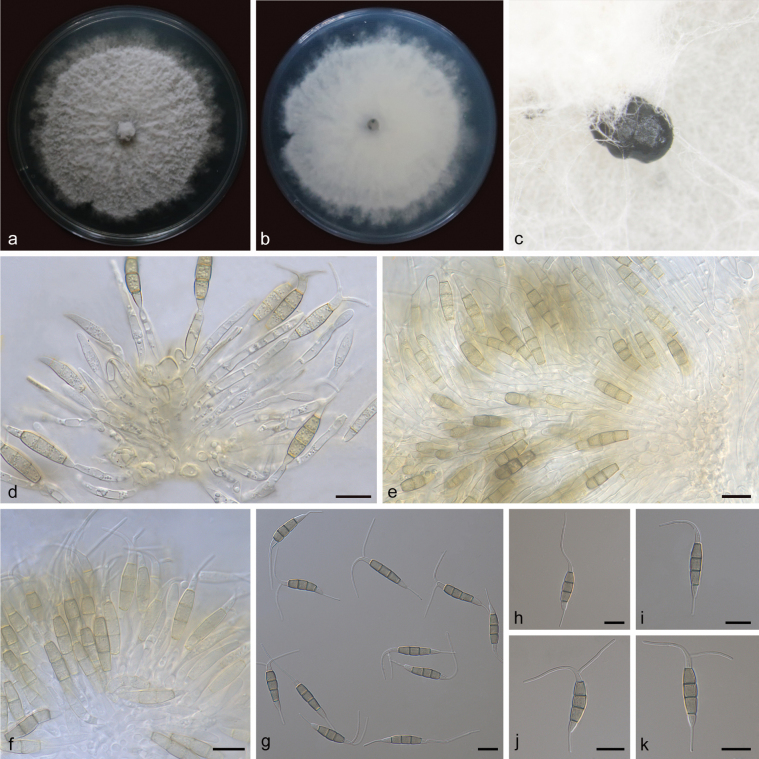
*Pestalotiopsisnannuoensis* (holotype: HMAS 352668) **a, b** the front and back of the colony after 14 days of culture on PDA **c** conidiomata on PDA **d–f** conidiophores and conidiogenous cells **g–k** conidia. Scale bars: 10 μm (**d–k**).

##### Etymology.

Referring to the collection site of the holotype, Nannuo Mountain.

##### Description.

Conidiomata in culture on PDA, 750–900 µm diam, subsphaeroidal, solitary, black conidial masses permeated above the mycelium. Conidiophores mostly degenerated into conidiogenous cells, hyaline, simple. Conidiogenous cells oval, hyaline, rough, aggregative, 10.6–19.4 × 2.2–3.4 μm. Conidia fusiform or subcylindrical, straight or slightly curved, 4-septate, 21.7–27.2 × 3.6–5.0 μm; basal cell conical, hyaline, rough, thin-walled, 3.9–5.4 µm; three median cells subcylindrical, brown, rough, thick-walled, the first median cell from base 4.4–6.2 μm, the second median cell 4.1–5.3 μm, the median third cell 4.5–5.7 μm, together 13.0–17.2 μm; apical cell conical or subcylindrical, hyaline, smooth, thin-walled, 2.9–4.6 µm; basal appendage tubular, single, centric, straight or slightly bent, unbranched, 6.8–9.2 µm; apical appendages tubular, 1–2, straight or bent, unbranched, 15.6–26.2 μm. Sexual morph not observed.

##### Culture characteristics.

After 7 days of dark cultivation at 25 °C on PDA, the colony diameter reached 75 mm, and the growth rate is 9.5–11.5 mm/day. Colonies filamentous to circular, with filiform margin, aerial mycelium on surface rugged, white, dense, fruiting bodies black; reverse white.

##### Additional specimen examined.

China, Yunnan Province, Menghai County, Nannuo Mountain, from rotted leaves, 18 Mar 2023, C.Z. Yin, Z.X. Zhang and X.G. Zhang, living culture SAUCC232204.

##### Notes.

*Pestalotiopsisnannuoensis* sp. nov. formed an independent clade (ML/BI = 100/1) in the phylogenetic tree based on ITS, *tub2* and *tef1α*, and was closely related to *P.diversiseta*. *P.nannuoensis* was different from *P.diversiseta* by 46/508 bp in ITS, 83/529 bp in *tub2*, and 59/465 bp in *tef1α*. Morphologically, *P.nannuoensis* was different from *P.diversiseta* by having shorter and thinner conidia (*P.nannuoensis*: 21.7–27.2 × 3.6–5.0 vs. *P.diversiseta*: 27.0–34.0 × 5.5–8.0 µm), and the number of apical appendages (*P.nannuoensis*: 1–2 vs. *P.diversiseta*: 3–5). (Maharachchikumbura et al. 2012). Therefore, *Pestalotiopsisnannuoensis* was identified as a new species of *Pestalotiopsis* by morphological and phylogenetic comparison.

#### ﻿ Pestalotiopsis rhaphiolepidis

Taxon classificationFungiAmphisphaerialesSporocadaceae

C.Z. Yin, Z.X. Zhang & X.G. Zhang sp. nov.

87FDE561-B431-5C5D-AA1C-E36DF37093B7

 851281

Fig. 4

##### Type.

China, Hainan Province, Jianfeng Town (18°42'35"N, 108°52'35"E), from diseased leaves of *Rhaphiolepisindica*, 11 Apr 2023, C.Z. Yin, Z.X. Zhang and X.G. Zhang, holotype HMAS 352669, ex-type living culture SAUCC367701.

**Figure 4.**
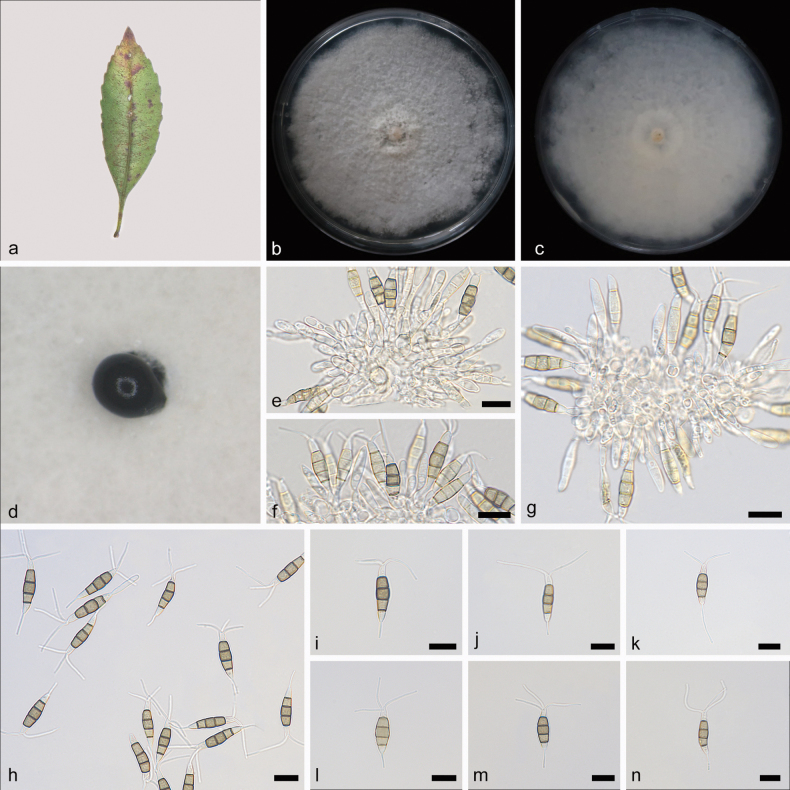
*Pestalotiopsisrhaphiolepidis* (holotype: HMAS 352669) **a** leaves of host *Rhaphiolepisindica***b, c** the front and back of the colony after 14 days of culture on PDA **d** conidiomata on PDA **e–g** conidiogenous cells and conidia **h–n** conidia. Scale bars: 10 μm (**e–n**).

##### Etymology.

Referring to the name of the host plant *Rhaphiolepisindica*.

##### Description.

Conidiomata in culture on PDA, 600–1000 µm diam, globular, solitary, black conidial masses permeated above the mycelium. Conidiophores mostly degenerated into conidiogenous cells, simple, hyaline. Conidiogenous cells fusiform, rough, discrete, 9.8–17.1 × 2.4–3.3 μm. Conidia fusiform, straight or slightly curved, 4-septate, 18.0–23.1 × 3.8–5.1 μm; basal cell conical, hyaline, rough, thin-walled, 3.3–5.1 µm; three median cells subcylindrical, light brown or brown, rough, thick-walled, the first median cell from base 3.0–4.7 μm, the second median cell 3.4–5.3 μm, the third median cell 3.7–5.6 μm, together 10.1–15.6 μm; apical cell subcylindrical or conical, hyaline, smooth, thin-walled, 2.8–4.7 µm; basal appendage tubular, single, centric, straight or slightly bent, unbranched, 4.7–9.8 µm; apical appendages tubular, 2–3, straight or bent, unbranched, 5.2–18.5 μm. Sexual morph not observed.

##### Culture characteristics.

After 7 days of dark cultivation at 25 °C on PDA, the colony diameter reached 90 mm, and the growth rate is 11.8–13.5 mm/day. Colonies filamentous to circular, flat, center raised, aerial mycelium on surface, with irregular edges, white, medium dense, fruiting bodies black; reverse white, multilayer rings from the middle to the edge.

##### Additional specimen examined.

China, Hainan Province, Jianfeng Town, from diseased leaves of *Rhaphiolepisindica*, 11 Apr 2023, C.Z. Yin, Z.X. Zhang and X.G. Zhang, living culture SAUCC367702.

##### Notes.

According to phylogenetic trees based on ITS, *tub2* and *tef1α*, *Pestalotiopsisrhaphiolepidis* sp. nov. was closely related to *P.inflexa* in a well support branch (ML/BI = 98/1). *P.rhaphiolepidis* was different from *P.inflexa* by 9/508 bp in ITS, 30/529 bp in *tub2*, and 16/465 bp in *tef1α*. Morphologically, *P.rhaphiolepidis* was different from *P.inflexa* by having shorter and thinner conidia (*P.rhaphiolepidis*: 18.0–23.1 × 3.8–5.1 vs. *P.inflexa*: 24.0–31.0 × 6.0–9.0 µm) and shorter apical appendages (*P.rhaphiolepidis*: 5.2–18.5 vs. *P.inflexa*: 20.0–30.0 μm) (Maharachchikumbura et al. 2011). Therefore, *Pestalotiopsisrhaphiolepidis* was identified as a new species of *Pestalotiopsis* by morphological and phylogenetic comparison.

## ﻿Discussion

*Pestalotiopsis* fungi are widely distributed and have been found all over the world, with 12,072 samples and 59,207 sequences were included in the GlobalFungi database (https://globalfungi.com/, accessed on 26 Jun 2024; Asia, 58.81%, North America, 20.84%, Europe, 5.86%, Africa, 5.38%, South America, 4.49%, Australia, 3.59%, Pacific Ocean, 0.78%, Atlantic Ocean, 0.21%, Antarctica, 0.05%). In this study, we obtained six strains of *Pestalotiopsis* from diseased and rotted leaves collected from Yunnan and Hainan Provinces in China. Based on phylogenetic analysis and morphological characteristics, we identified six strains as three new species of *Pestalotiopsis*, *P.aporosae-dioicae*, *P.nannuoensis* and *P.rhaphiolepidis*. It is worth noting that the plant hosts of *Pestalotiopsis* fungi are abundant, such as Theaceae, Arecaceae, and Fagaceae (Maharachchikumbura et al. 2014; Jiang et al. 2022b). We first reported the new hosts of *Aporosadioica* (Phyllanthaceae) and *Rhaphiolepisindica* (Rosaceae) by identifying the *Pestalotiopsis* fungi. This suggests that there were more potential new species of *Pestalotiopsis* to be discovered in these two host plants. *Pestalotiopsisnannuoensis* was found on rotted leaves and its host is unknown. *Pestalotiopsis* fungi are mostly plant pathogens, and the relationship between the three newly discovered species and their hosts and their effects on cash crops needs further study (Zhang et al. 2012a; Maharachchikumbura et al. 2013; Jayawardena et al. 2016; Liu et al. 2017; Yang et al. 2017; Diogo et al. 2021; Prasannath et al. 2021).

Since Steyaert introduced *Pestalotiopsis* into Sporocadaceae (Amphisphaeriales, Ascomycota) in 1949, more and more species of *Pestalotiopsis* have been discovered (Steyaert 1949; Akinsanmi et al. 2017; Liu et al. 2017; Nozawa et al. 2017; Ariyawansa and Hyde 2018; Jiang et al. 2018; Tibpromma et al. 2018; Tsai et al. 2018). However, due to the similarity of the spore structure, the classification of *Pestalotiopsis* is unclear, and the traditional identification method was very complicated work. With the development of molecular technology, the identification method combining morphology and phylogeny has been accepted by more and more taxonomists. Maharachchikumbura et al. (2014) applied phylogenetic analysis in the classification of *Pestalotiopsis* to make the classification more clearly. Therefore, phylogenetic analyses based on ITS, *tub2* and *tef1α*, including maximum likelihood (ML) and Bayesian inference (BI), have been widely used. Meanwhile, the size of spores, length, position, origin and number of branches of apical appendages and basal appendages are important for the classification of *Pestalotiopsis* fungi. Taking several subjects in this study as examples, *Pestalotiopsisaporosae-dioicae* and *P.rhaphiolepidis* can be identified as new species by morphological comparison with other species in a well-supported clade (Maharachchikumbura et al. 2011, 2014). Although *Pestalotiopsisnannuoensis* was an independent clade, it had the basic characteristics of *Pestalotiopsis*, and was significantly different from several closely related species in spore size and number of apical appendages, so it was also identified as a new species (Maharachchikumbura et al. 2012). Based on the results of this study, we believe that we will isolate more potential *Pestalotiopsis* fungi in the future. With the development of biotechnology and the deepening of the research on *Pestalotiopsis* fungi, it has become an important research focus and direction to sequence the genome of *Pestalotiopsis* fungi, annotate its structure and function, and explore the types and applications of secondary metabolites.

## Supplementary Material

XML Treatment for Pestalotiopsis aporosae-dioicae

XML Treatment for Pestalotiopsis nannuoensis

XML Treatment for Pestalotiopsis rhaphiolepidis

## ﻿Additional information

### Author ORCIDs

Changzhun Yin https://orcid.org/0009-0000-0034-2199

Zhaoxue Zhang https://orcid.org/0000-0002-4824-9716

### Data availability

All of the data that support the findings of this study are available in the main text or Supplementary Information.

## Supplementary materials

Supplementary material 1 Original and spliced sequences of three genes of all strains of the genus *Pestalotiopsis* for phylogenetic analysis Data type zip https://binary.pensoft.net/file/1089689This dataset is made available under the Open Database License (http://opendatacommons.org/licenses/odbl/1.0/). The Open Database License (ODbL) is a license agreement intended to allow users to freely share, modify, and use this Dataset while maintaining this same freedom for others, provided that the original source and author(s) are credited.Changzhun Yin, Zhaoxue Zhang, Shi Wang, Liguo Ma, Xiuguo Zhang
